# A Non-Invasive Soil Moisture Sensing System Electronic Architecture: A Real Environment Assessment

**DOI:** 10.3390/s20216147

**Published:** 2020-10-29

**Authors:** Leonardo Franceschelli, Annachiara Berardinelli, Marco Crescentini, Eleonora Iaccheri, Marco Tartagni, Luigi Ragni

**Affiliations:** 1 Department of Electrical, Electronic and Information Engineering “Guglielmo Marconi”—University of Bologna, Via dell’Università, 50, 47521 Cesena, Italy; m.crescentini@unibo.it (M.C.); marco.tartagni@unibo.it (M.T.); 2 Department of Industrial Engineering, University of Trento, Via Sommarive, 9, 38123 Povo, Italy; annachiara.berardinelli@unitn.it; 3 Centre Agriculture Food Environment, University of Trento, Via E. Mach, 1, 38010 S. Michele all’Adige, Italy; 4 Department of Agricultural and Food Sciences, Alma Mater Studiorum, University of Bologna, Piazza Goidanich 60, 47521 Cesena, Italy; eleonora.iaccheri4@unibo.it (E.I.); luigi.ragni@unibo.it (L.R.); 5 Interdepartmental Center for Industrial Agri-Food Research, University of Bologna, Via Q. Bucci 336, 47521 Cesena, Italy

**Keywords:** soil moisture, silty clay loam, waveguide spectroscopy, “gain” and “phase”, partial least square regression (PLS)

## Abstract

This paper will show the electronic architecture of a portable and non-invasive soil moisture system based on an open rectangular waveguide. The spectral information, measured in the range of 1.5–2.7 GHz, is elaborated on by an embedded predictive model, based on a partial least squares (PLS) regression tool, for the estimation of the soil moisture (%) in a real environment. The proposed system is composed of a waveguide, containing Tx and Rx antennas, and an electronic circuit driven by a microcontroller (MCU). It will be shown how the system provides a useful and fast estimation of moisture on a silty clay loam soil characterized by a moisture range of about 9% to 32% and a soil temperature ranging from about 8 °C and 18 °C. Using the PLS approach, the moisture content can be predicted with an R^2^ value of 0.892, a root mean square error (RMSE) of 1.0%, and a residual prediction deviation (RPD) of 4.3. The results prove that it is possible to make accurate and rapid moisture assessments without the use of invasive electrodes, as currently employed by state-of-the-art approaches.

## 1. Introduction

In the last decade, the indirect techniques for soil moisture assessment have become increasingly important as alternative tools to the standard time-consuming thermo-gravimetric method [1]. These techniques are based on the assessment of physical and chemical soil properties and are crucial in the new “precision agriculture” application field. Among these, a considerable part of literature is focused on the inference of moisture on soil dielectric properties [2], variables that describe the electric polarization of the matter when subject to an external electric field. The complex (or apparent) relative dielectric permittivity is defined by real and imaginary components. The real component accounts mainly for energy stored in the system owing to the alignment of dipoles with the electromagnetic field. Usually, applications in active and passive remote sensing use the change of this part of the dielectric constant because it contains most of the information [3]. The imaginary component (related to dielectric loss factor) is mainly due to molecular relaxation and accounts for energy dissipation effects [1]. Even if a smaller part of the information resides in there, it could be utilized to boost the sensing performance [4]. In addition to the temperature and frequency of the electromagnetic field [4,5], dielectric properties are also a function of parameters such as moisture content [6], bulk density [7], and soil constituents [5], which could thus be described thanks to a dielectric characterization, making it a fundamental technique for indirect soil sensing methods such the one presented in this paper.

Two main measuring techniques have been developed based on soil dielectric properties, time domain reflectometry (TDR) and frequency domain reflectometry (FDR). Both are based on the fact that, within the range 100 MHz–2 GHz, the apparent relative dielectric permittivity of soil is chiefly affected by the apparent relative dielectric permittivity of water ($\varepsilon {{}^{\prime }}_{water}\approx 80$ at 20 °C), as it is much greater than air ($\varepsilon {{}^{\prime }}_{air}=1$) and solid particles ($\varepsilon {{}^{\prime }}_{soil}\approx 3-7$). Thus, within this frequency range, it is possible to correlate soil relative dielectric permittivity with soil moisture through a calibration curve. Moreover, at higher frequencies, it becomes almost invariant with respect to the frequency; under such a condition, this medium property can be referred to as the apparent dielectric constant of soil [1].

TDR determines the dielectric constant by measuring the propagation time of electromagnetic waves, sent from a pulse generator of a tester whose probe is immersed in the soil. Electromagnetic waves propagate through the coaxial cable to a rod-shaped TDR probe, made of stainless steel or brass. Part of the incident electromagnetic wave is reflected at the beginning of the probe because of the impedance difference between the cable and the probe. The wave propagates through the probe until it reaches the end of it, where it is reflected. The round-trip time of the wave from the beginning to the end of the probe is directly related to the dielectric constant [8,9]. The moisture content is estimated from the dielectric constant thanks to regression models based on third-order polynomial equations [10] or with additional parameters describing the soil physical characteristics [11]. Calibration equations are specific for soil typology and properties, so different curves should be acquired. Different calibration equations are requested when the soil typology and properties are varied. Water, air distribution, solid particles, and its orientation affect the reflections, because apparent dielectric permittivity measured in the time domain depends on both parts of complex dielectric permittivity [12]. Moreover, saline soils are difficult to measure [13].

Other techniques consider soil as the dielectric of a capacitor. FDR sensors use the interaction of the fringing field of capacitance sensors with the surrounding soil, whereby the capacitance is influenced by the soil bulk electrical permittivity and thus by soil moisture content [14]. The frequency of oscillation decreases with increasing moisture contents. Nowadays, major technical progress is achieved using FDR, thanks to its lower costs in terms of both time and financial resources, compared with the TDR technique. However, FDR is sensitive to the composition of the region near the probe and to the general clay content and type, causing a worsening in accuracy and precision of the soil moisture measurements [15]. Moreover, both techniques present a problem related to the probe: a non-perfect adhesion with soil induces air interface effects in the measurement, biasing the results [9,15].

Several attempts were made to develop non-invasive systems based on microwave reflection, especially in the spectral range of near-infrared (NIR), exploiting multivariate statistical algorithms to predict moisture values. Some applications were created to be used in movement, attached to the subsoiler chisel or shank of a tractor [16,17], whereas the majority of them were immobile, directly in contact with the soil or just above it. They are based on different technologies: LEDs [18], optical units [19], antennas [20,21], bistatic scatterometer [22], and waveguide [4,23]. Finally, some works focus more on the application and comparison of different statistical analysis, using existing spectrometers to acquire data [24]. Table 1 shows a comparison among the principal results of these systems.

In a previous work [4], we explored the potentiality of a non-invasive technique based on an open-ended waveguide for the prediction of the soil moisture content, starting from “gain” (defined as the ratio between the power of the received and the emitted waves) and “phase” (defined as the difference of phase between received and the emitted waves) spectral data acquired on samples of different types of soils in controlled lab conditions. The results, obtained in combination with partial least squares regression (PLSR) and multi-way PLS tools, showed that a different moisture content (%) in the soil involves changes in both “gain” and “phase” waveforms; predictive models of the moisture content (%) were characterized by R^2^ values up to 0.959 (segmented cross validation) for silty clay loam soil type [4]. In another work, standard PLSR and derived analysis (K-OPLS) are applied to measurements done with a dipole antenna, obtaining R^2^ values up to 0.964 for silty clay loam [21].

With PLSR, it is possible to create a calibration model from the “gain” and “phase” data measured by the system, then the calibration coefficients are used to implement the calculation of the moisture value directly in the device microprocessor. The proposed system, in combination with multivariate data analysis, can be considered an innovative approach compared with the traditional techniques, avoiding invasive probes, being independent from a data post-processing, and having good accuracy, thanks to the proposed statistical tools.

The purpose of this work is as follows: (i) To show a compact and fast sensing architecture approach based on the microwave impedance spectroscopy and multivariate analysis. Therefore, the measurements are made using a compact electronic architecture in a real environment and not by calibrated instruments in laboratory conditions; (ii) To assess the architecture and the model on a real soil environment, as opposed to the previous works [4,24]: in both papers, “gain” and “phase” data were obtained (with the same system and a dipole antenna, respectively) and then analyzed with multivariate statistical analysis, but starting from soil samples prepared in laboratory, in controlled conditions and with a well-defined stratification.

## 2. Approach and Architecture

### 2.1. Physical Sensing Approach

**Figure 1:**
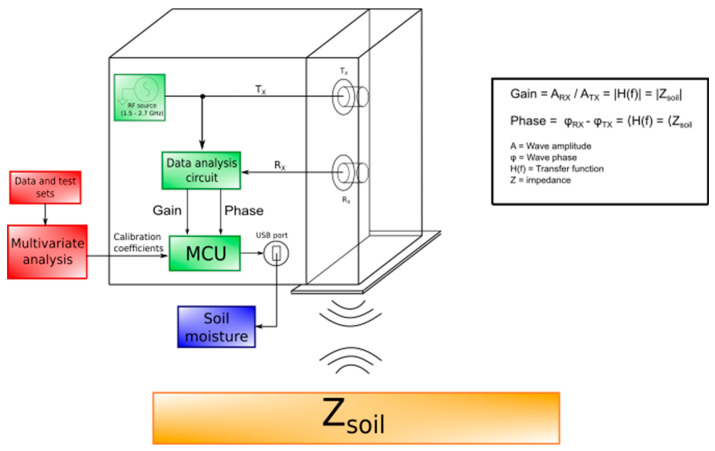
System general functioning. A data analysis circuit calculate “gain” and “phase” data, using as input transmitted and received electromagnetic waves. These data are used to calculate the soil moisture directly in the microcontroller unit (MCU), thanks to calibration coefficients obtained from a multivariate statistical analysis.

The main components of the system are shown in Figure 1. It could be divided into two fundamental parts: an open rectangular waveguide (96 mm × 245 mm × 46 mm), containing Tx and Rx antennas, and a plastic box (200 mm × 250 mm × 100 mm) containing the electronic circuit, descripted in a more detailed way in Section 2.3. Figure 1 also highlights the main components involved in the system general functioning: a series of electromagnetic waves are created by a Radio Frequency (RF) source, and then transmitted to the soil through the open-ended waveguide. The reflected waves are read by the Rx antenna and used as input for a data analysis circuit to obtain “gain” and “phase” data. These are multiplied in the microcontroller unit (MCU) with calibration coefficients calculated using multivariate analysis to obtain soil moisture estimation. To measure this parameter, the system exploits the concept of impedance spectroscopy, an indirect method based on physical interactions between the matter under test and an electromagnetic excitation. At the interface between the two different materials, air and soil, rapid variations of physical and electric properties cause refraction and reflection phenomena in the wave propagation.

The transmitted and reflected waves can be described, respectively, as follows:

(1) $$Tx\left(t\right)=\;A_{Tx}e^{j\varphi_{Tx}}e^{j2\pi ft}$$

(2) $$Rx\left(t\right)=\;H\left(f\right)A_{Tx}e^{j\varphi_{Rx}}e^{j2\pi ft}=\;A_{Rx}e^{j\varphi_{Rx}}e^{j2\pi ft}$$

where *A* is the wave amplitude; *j* is the imaginary unit; f is the frequency; *t* is the time; φ is the phase; and *H*(*f*) is the transfer function between x and y, represented by the soil impedance [25].

It is possible to see that the relationship between the amplitudes and the phases of the two signals are as follows:

(3) $$A_{Rx}=\;\left|H\left(f\right)\right|A_{Tx}\to \frac{A_{Rx}}{A_{Tx}}=\;\left|H\left(f\right)\right|=|Z_{soil}$$

(4) $$\;\varphi_{Rx}=\langle H\left(f\right)+\;\varphi_{Tx}\;\to \;\varphi_{Rx}-\;\varphi_{Tx}=\langle H\left(f\right)=\langle Z_{soil}$$

These equations explain that the reflection of an electromagnetic wave is still a wave, with the same frequency of the transmitted one, but with a different amplitude value and phase shift. H(f) is determined by the ratio between *A_Rx_* and *A_Tx_*, called “gain”, and the difference between *φ_Rx_* and *φ_Tx_*, called “phase”. Then, the soil moisture value is extracted from the impedance information, thanks to a predictive model based on multivariate statistical analysis, as detailed in Section 2.2.

The frequency range of electromagnetic waves was chosen to be 1.5–2.7 GHz, maintaining the same range that gave very good results in a previous work [4]; in this spectral region, it is possible to obtain information about soil moisture and, at the same time, design the whole system in a practical and compact way. The waveguide dimensions (96 mm × 245 mm × 46 mm) were chosen to obtain a cut-off frequency equal to 1.56 GHz.

### 2.2. Predictive Model

A predictive model allows to estimate moisture values from gain and phase variations. This model was calculated thanks to a multivariate statistical analysis called partial least square regression (PLSR), developed by Wold in the late 1960s [26].

Following multivariate analysis, assume we have *K* variables associated with *N* observations (or objects). Therefore, an observation is described by a point in a K-space. In our case, each observation is an acquired spectrum and variables are magnitude or phase response at different wavelength and soil moisture. From the experimental point of view, for each observation, we can measure both the “gain” and “phase” spectral data, with the proposed system, and the real soil moisture, with a more traditional (and invasive) technique called “thermo-gravimetrical method”, described in more detail in Section 2.5.

This preliminary phase is intended to acquire the so-called *calibration dataset*. The first approach is to understand the main relationships between all variables, with principal component analysis (PCA). PCA could show hidden relationships between variables of observations. More specifically, we can identify A < K variables, called *principal components* (PC), summarizing the most effective relationships between variables among observations. From a geometrical point of view, we can have a “good summary” of the observations in an *A*-dimensional subspace, smaller than the original *K*-dimensional one. The A-space is generated by the orthogonal principal components that better describe the inter-relationships between observations.

PLSR follows this approach. Not all the variables are equal; some are always available (magnitude and phase for each wavelength) and others (moisture) are not always available. This means that our system is always able to determine a spectrum, but we could not have the availability of the soil moisture (e.g., taken from an external instrument). Therefore, in a sensing approach, we would like to determine the unknown moisture from the known spectrum. Therefore, PLSR determines *M output or dependent variables Y* (in our case M=1, soil moisture) thanks to the construction of a *model* to *predict* this variable from spectrum. The idea is to collect spectra (*X* calibration data set) from determined moisture values (*Y* calibration data set) and develop a model to predict unknown soil moisture from acquired calibrating spectra.

In PLSR, an X matrix of dimensions N × K (in our case, this is the collection of *N* spectra, where each of them is composed of *K* amplitude (or phase) responses) and a *score* matrix ***T*** of dimension N × A (where A < K is the number of principal components), formed by the ***X*** directions with maximum variance, are defined. In other terms, using regression analysis terms, ***T*** is a good summary of ***X*** so that the error (residuals) is small. In matrix notation, it is as follows:

(5) $$T=XW\to X={TW}^{{}^{\prime }}+E$$

where ***W*** (dimensions *K* × *A*) is the weight (or *loadings*) matrix and ***E*** is the residual matrix. However, it is not guaranteed that T contains the information needed to best predict Y; this information could be in other principal components (PC) with less variance [26,27].

Therefore, PLSR identifies even better directions in the score subspace, called *latent variables* (LV), which maximizes the variance of the output variable ***Y***. This could be summarized by saying that ***X***-scores (***T*** matrix) are also good predictors of ***Y*** (dimensions *N × M*):

***Y*** = ***TC′*** + ***F*** (6)

where ***F*** is a residuals matrix. Thanks to the maximization of Y variance, it is possible to obtain a good prediction model even if spectra do not have great variations between them (max difference in gain spectra equal to 1.65 dB, in the present work) [28,29,30,31]. The previous equation could be easily interpreted from the geometric point of view: ***T*** is an *A*-dimension subspace in the *K*-dimension space of ***X***; therefore, ***C*** identifies the best direction in the subspace of X that also has the maximum variance respect to the output.

Therefore, we can describe the output using the calibration data set as follows:

***Y*** = ***TC’*** + ***F*** = ***XWC′*** + ***F*** = ***XB*** + ***F*** (7)

The goal of PLSR is to estimate, using several iterating techniques, the matrix ***B*** (*K x 1*), thus allowing to use the linear model,

(8) $$\hat{Y}=XB$$

to calculate ***Y*** values directly from new ***X*** data [32]. This model is what will be easily embedded in our system for a fast estimation of moisture.

**Figure 2:**
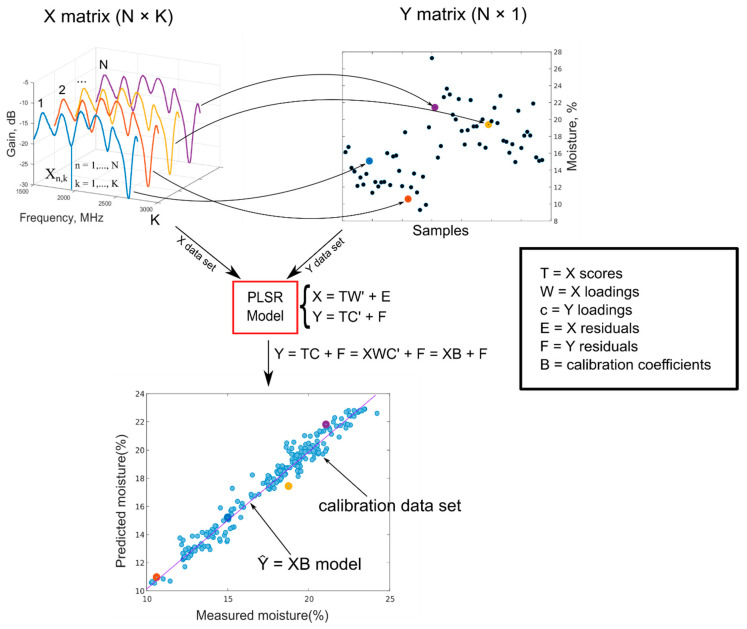
Partial least square regression (PLSR). The upper-left plot represents “gain” spectra obtained with N different acquisitions (X data set), each of these linked to a soil moisture value represented in the upper-right plot (Y data set). These two data sets are used as input for the creation of a PLSR model, which allows to predict soil moisture values from spectral data. The lower plot shows the relation between the moisture values measured with the traditional thermo-gravimetrical method (*x*-axis) and predicted with the PLSR model (*y*-axis). Different colors are used to highlights the same acquisitions in the three plots.

A summary and concise scheme of the whole analysis is shown in Figure 2.

### 2.3. Electronic Implementation and Architecture

The measurement system is composed of three principal components: a data-control and elaboration system, an RF source, and a gain-phase detector.

The first consists of a microcontroller, a D/A converter, and a serial–USB converter. The selected microcontroller is a MICROCHIP PIC24FJ256GB606. It manages the measurement process and the communication with the serial interface (UART/USB converter cable). This microcontroller has a few interesting features, in particular, a SRAM data storage with a capacity of 32 Kbytes and with 16-bit addresses, making PIC24F the ideal microcontroller for data-logging applications of significant amounts of data. Moreover, the PIC presents a Harvard modified architecture; that is, the data, normally present inside the RAM memory, can also be saved in the program memory, with the possibility to remap a portion of the latter in the data memory (program space visibility, PSV).

The D/A converter is a 16-bit Analog Devices AD5761R. It connects the MCU with the RF source, translating the digital value provided by the microcontroller into an analog voltage thanks to a voltage ramp from 0 to 20 V, approximately.

It has a resolution of 16-bit, a unipolar or bipolar output, an output noise equal to 35 nV/Hz, a maximum integral nonlinearity (INL) of ±2 Least Significant Bit (LSB), and a maximum output settling time of 12.5 μs (with a step equal to 20 V).

The RF source consists of a VCO MiniCircuits ZX95-2700A; it translates the output voltage of the DAC into an ideally sinusoidal wave at a frequency dependent on the input voltage. Its most interesting features are the frequency of the wave generated from 1.5 GHz to 2.7 GHz (compatible with the project specifications), the typical output power of 3.3 dBm, the low phase noise, the tuning voltage from 0.15 V to 25 V, the supply voltage of 5 V, and the maximum power supply current of 35 mA. The signal coming out of the VCO has a power of around 4 dBm at maximum, which is too low compared with the design specifications, so it is necessary to perform an amplification to reach the minimum transmitted power through an RF amplifier circuit. The chosen component is the QORVO TQL9092, an ultra-low noise amplifier (LNA) with operating band from 0.6 to 4.2 GHz.

Finally, the signal amplified by the LNA is supplied to the rectangular guide, which generates waves and collects the reflected ones, thanks to a Tx and a Rx antenna.

The gain phase detector compares the transmitted and reflected waves and provides the measured information to the microcontroller.

**Figure 3:**
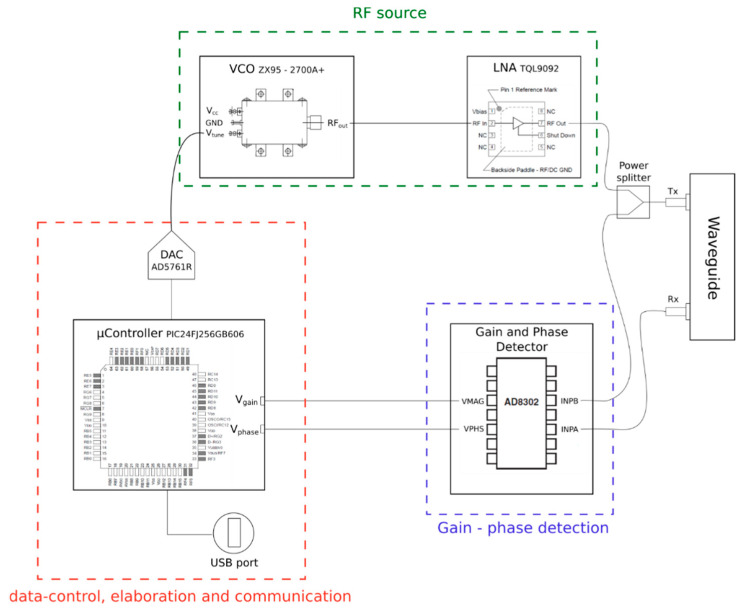
Electronic system layout.

The characteristics, measured by the instrument, are in the range of −30/+30 [dB], with a scale of 30 mV/dB for the gain, and 0–180° with a scale of 10 mV/° for the phase. The gain and phase output voltages vary in a range from 0 V to 1.8 V. In addition, the component provides a reference voltage of 1.8 V, which serves as a full scale for the output voltages. A layout of the electronic systems is shown in Figure 3.

### 2.4. Embedded Model Approach

**Figure 4:**
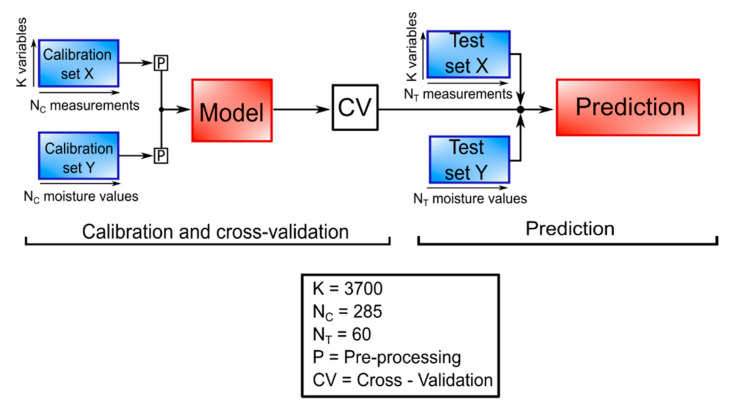
PLSR scheme.

A software called PLS_Toolbox [33] was used for the creation and the validation of the model. As shown in Figure 4, the process is divided into three fundamentals steps: model creation, cross-validation, and validation.

The two calibration sets were created with the measurements done on the soil (X, independent variables) and with the moisture values calculated with the thermo-gravimetric method (Y, dependent variables). Before the model creation, an autoscale pre-processing was applied to both X and Y data sets, consisting of mean centering and scaling each variable to unit standard deviation.

The algorithm used for the model calculation is an evolution of the original one, called SIMPLS. The main difference between the two is that, in SIMPLS, the deflation process does not apply to the starting data matrices X_0_ and Y_0_, but to the cross-product S_0_, resulting in faster computation and less memory requirements. Secondly, all factors are equally easy to interpret, namely as simple linear combinations of the original variables [34].

For cross-validation, a method called “venetian blinds” was used: each subset is determined by the selection of every nth object in the data set, starting at objects numbered 1 through s. This method is simple and easy to implement, and generally safe to use if there are relatively many objects already in random order [35]. The parameter root mean square error of cross validation (RMSECV) was used for the selection of the number of latent variables, done automatically by the software thanks to a function called “choosecomp”. The optimal number is the one that allows a good equilibrium between generalization and minimization of RMSECV error [36].

Another algorithm, already implemented in the software, was used to select only the useful X variables, to improve the regression model. According to this algorithm, in the first run, the variables with the lowest VIP (variable importance in projection) values are eliminated. If the model improves, this is repeated until convergence [37]. Finally, new measurements were used to create X and Y test sets. With these, a validation of the model was performed, obtaining the statistical parameters R^2^, RMSE, and residual prediction deviation (RPD) for prediction.

Calibration coefficients are obtained from the refined model, resulting in an array ***A*** and a single offset value ***B***, with which it is possible to calculate the soil moisture value ***Y*** directly from a new measurement X, with the following formula:

(9) $$\hat{Y}=XA+B$$

**Figure 5:**
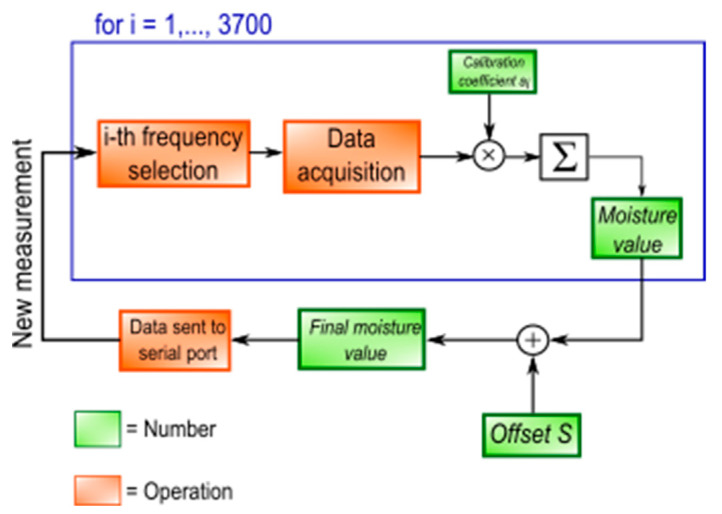
Moisture calculation in the MCU.

One of the main purposes of this work is to obtain a stand-alone system, so the moisture calculation is executed in the microprocessor, concurrently to the gain and phase acquisition; for each frequency, these values are multiplied by the corresponding coefficient, and summed up with the previous multiplication in a new variable. At the end of the whole acquisition, an offset B *(1 × 1)*, also calculated by PLSR, is summed to this variable, obtaining the final moisture value, sent to the PC through a serial port. The whole process is shown in Figure 5.

The PSV function of the PIC24F is used to save all the coefficients in the program memory, in order to speed up the moisture calculation and take advantage of the bigger memory space.

Coefficients, originally decimal numbers, are multiplied by 100.000 and saved as int. The final moisture value is then divided by 100.000, obtaining a fast acquisition time (about 45 s) with a precision to the fifth decimal digit.

### 2.5. Data Acquisition

Spectral acquisitions were conducted on different areas of a silty clay loam soil located in the Romagna region (Italy). An average value of the soil bulk density of 1.40 ± 0.09 g/cm^3^ was calculated starting from nine measurements conducted on different soil areas. Measurements were done with a core drill machine, with a diameter of about 48 mm and a height of about 56 mm.

For each chosen location, three measurements were acquired, rotating the container with respect to its axis of an approximately constant angle (40°). For each acquisition, the system was placed in such a way that the longitudinal axis of the waveguide was normally oriented with respect to the soil surface; a good adhesion of the waveguide aperture to the soil was ensured after a brief cleaning of fallen leaves and other organic detritus that could alter the measure. The time requested to perform a single measurement is about 45 s.

The acquisitions were taken during October to November 2018, with a soil temperature range of 8.1–17.5 °C and an air temperature range of 7.7–20.9 °C.

A total of 345 measurements (115 soil areas × 3 acquisition per area) were obtained over these two months. Nine measurements on soil, together with nine others done on air, were used to assess the accuracy of the system, thanks to the calculation of standard deviation, coefficient of variation, and maximum difference. These parameters were obtained for each triplets of acquisition (carried out with different location on soil and temperature), for a total of three, and then mediated between them to obtain the final result.

**Figure 6:**
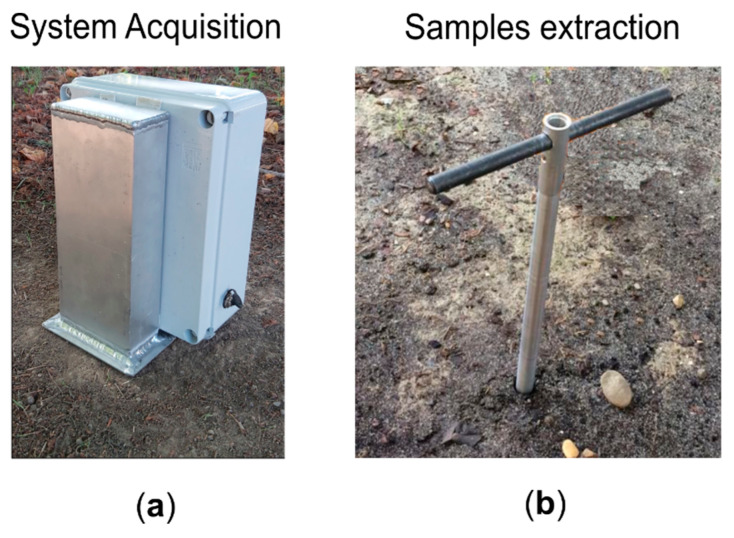
(**a**) System acquisition and (**b**) samples extraction on real soil.

After each triplet of acquisitions, a section of metallic tube was used to perform a coring process on the soil, obtaining cylindrical samples of about 10 cm and diameter of about 0.8 cm. Example photos of an acquisition and a sample coring are displayed in Figure 6.

For each sample, the moisture value (%) was evaluated with the thermo-gravimetrical method; each sample was weighted, then put in an oven at 105 °C for about 24 h and weighted again. The weights were used to find the gravimetric moisture values *θ_M_*, thanks to the following formula:

(10) $$\;\;\theta_{M}=\;\frac{\left(w_{w}+\;t_{a}\;\right)-\left(w_{d}+\;t_{a}\right)}{\left(w_{d}+t_{a}\right)-\;t_{a}}\;\times 100$$

The parameters *w_w_* and *w_d_* represent the masses of the soil before and after the day in the oven, respectively, and *t_a_* represents the tare mass [1].

In the end, the independent variables (X dataset) were arranged in a K = 3700 (variables, spectrum) × N = 345 (measurements) matrix, whereas a N = 345 (measurements) × M = 1 (variable, moisture content %) vector column was created for the dependent variable (Y dataset). Then, both datasets are split between calibration, with N_C_ = 285 measurements, and test, with N_T_ = 60 measurements.

## 3. Results and Discussion

Acquired samples of soil were characterized by a moisture content ranging from 9.3% to 31.7% and by a temperature from 8.1 °C to 17.5 °C. The acquisitions were distributed with respect to the temperature as follows: 14% in the range of 7 °C–11 °C, 18% in the range of 11°C–14 °C, and 68% in the range of 15 °C–17.5 °C. Hand-made soil samples with lower moisture values (in the range of 0.1–7.2%) were already tested in a previous work [4]; the PLSR model, created with spectra acquired on a total soil moisture range of about 0.1–28%, showed a good prediction ability, with an R^2^ value always higher than 0.92, for both single and double-layered samples.

**Figure 7:**
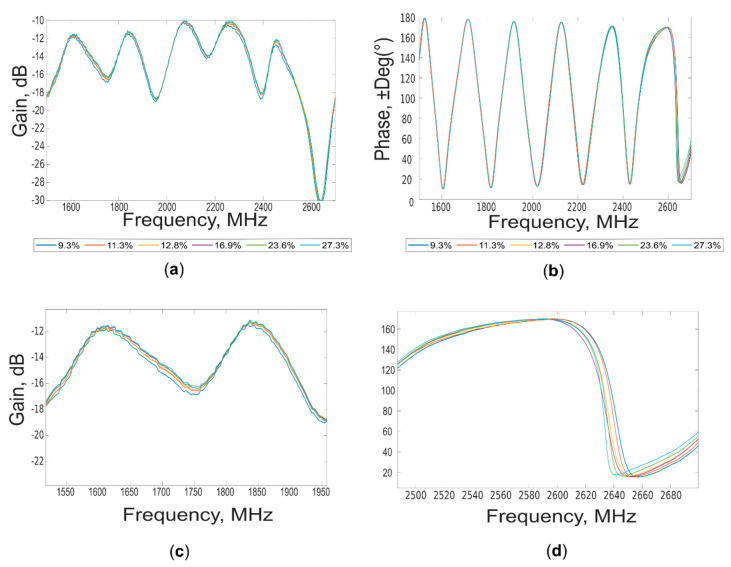
(**a**) “Gain” and (**b**) “phase” waveforms acquired at different moisture content (%). (**c**,**d**) Magnifications of the waveforms, in the regions where the spectral differences are more pronounced.

Examples of “gain” and “phase” waveforms acquired at different soil moisture contents (%) are shown in Figure 7.

Spectral differences can be appreciated during the entire range of the explored frequencies, especially for “gain” spectra (1.5 GHz–2.7 GHz), according to the moisture content. It appears evident the influence in the spectra of the complex water–soil chemical-physical interactions. These changes can be better visualized in the lower portion of the figure showing a magnification of both “gain” and “phase” in two different spectral ranges. For these acquisitions, a temperature of the soil ranging from 16.5 °C to 17.5 °C was measured. No evident loss of continuity in the waveforms was detected for the under cut-off sub frequency range (from 1.50 GHz to 1.56 GHz), where the emitted power appears enough to return information related to moisture content.

**Figure 8:**
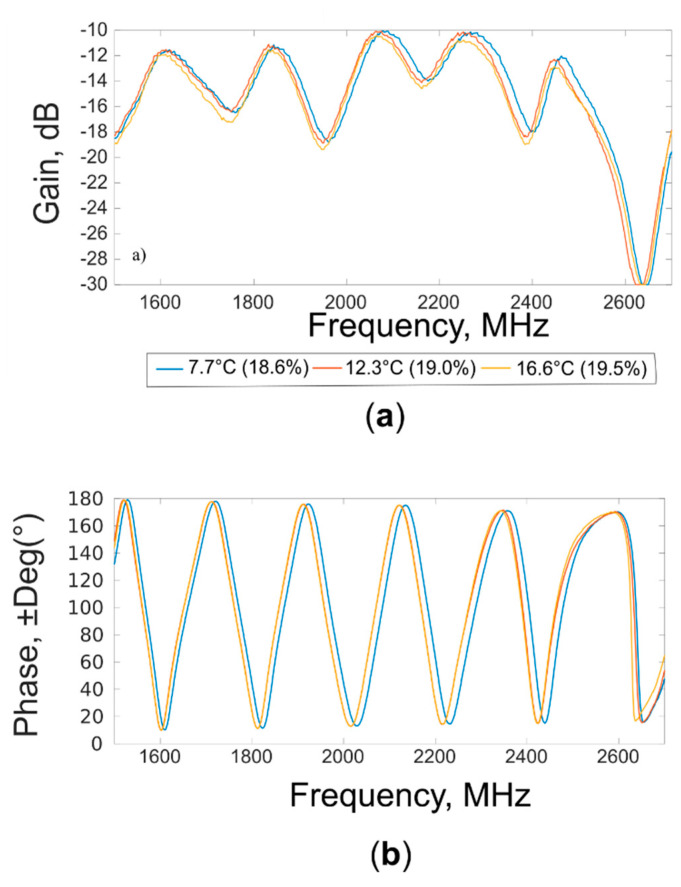
(**a**) “Gain” and (**b**) “phase” waveforms acquired at different soil temperatures (°C), at moisture of about 19.0%.

In order to understand the influence of the temperature on spectral acquisitions, other examples of “gain” and “phase” waveforms are shown in Figure 8.

The waveforms were acquired at soil temperatures of 7.7 °C, 12.3 °C, and 16.6 °C on soil samples characterized by small variation of moisture contents (18.6%, 19.0%, and 19.5%, respectively). Shifts of the waveforms are evident in different part of the spectrum for both “gain” and “phase”. These differences reflect the known dependence from the temperature of the loss factor and dielectric constant.

**Figure 9:**
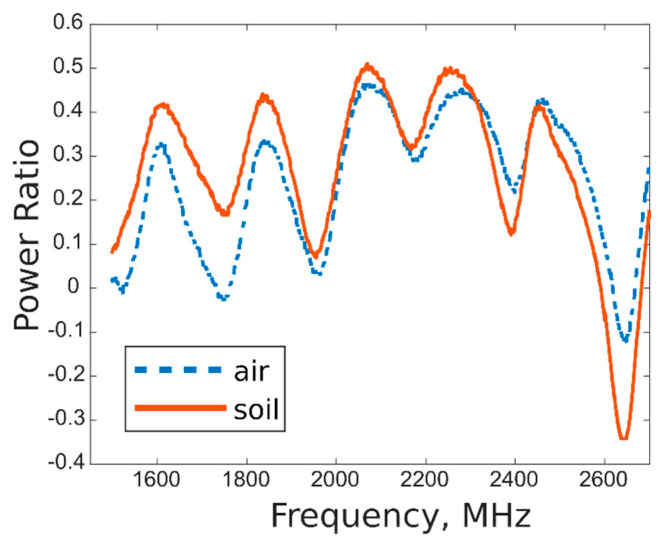
Characterization of mutual coupling between Tx and Rx antennas, for acquisitions on air and soil.

Moreover, the mutual coupling between the antennas was characterized, calculating the power emitted by the Tx antenna (which varies by the wave frequency, in a range of 20.4–22.5 dB) and subtracting from it the measured gain values, for the different frequency, to obtain the RX power. The coupling was obtained as ratio between Tx and Rx power, for acquisition taken on both air and soil, in order to compare the response when open-end waveguide radiated in open space and in soil. The results are shown in Figure 9; as expected, different trends were obtained for acquisition on air and on soil, evidencing the ability of the systems to well differentiate the two media, both remaining in an acceptable range of power ratio values.

The results of the PLS analysis conducted on “gain” and “phase” spectra for the prediction of the moisture content (%) are summarized in Table 2 in terms of R^2^, RMSE, and RPD values for calibration, segmented cross validation, and test set validation. The optimal numbers of latent variables were 6 for “gain” model and 5 for the “phase” one. The test sets were created with measurements and moisture values chosen from X and Y calibration sets (and thus not included in model creation). For every chosen sample, all three acquisitions were put in the test set, in order to avoid the presence of the same sample’s acquisitions in both data set. The samples (60 measurements, about 20% of the total) were randomly selected in order to cover all the moisture range of the data used for the calibration.

As expected from the waveform’s visual exploration, the best predictions were obtained using “gain” spectra. In test set validation, the moisture content can be predicted with an R^2^ value of 0.892, an RMSE of 1.0%, and an RPD of 4.33 with the gain model. For “phase”, an R^2^ value of 0.781, an RMSE of 1.8%, and an RPD of 2.4 were obtained.

**Figure 10:**
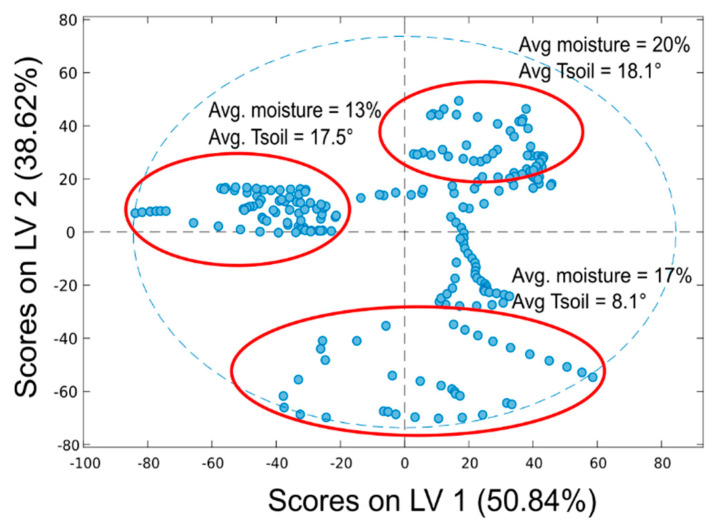
Scores plot for the PLS model in calibration obtained for “gain” spectra (first two latent variables (LVs)).

The scores plot for the model obtained from “gain” spectra (in calibration) is reported in Figure 10 for the first two latent variables (LV1 and LV2). This plot shows how much each measurement is influenced by the two LVs, allowing to understand which physical parameters they represent; that is, samples scores are distributed according to the moisture content (%) along the first latent variable, which accounts for 50.84% of the variance. The second latent variable (38.6% of the variance) appears to describe differences in the spectra due to temperature changes.

**Figure 11:**
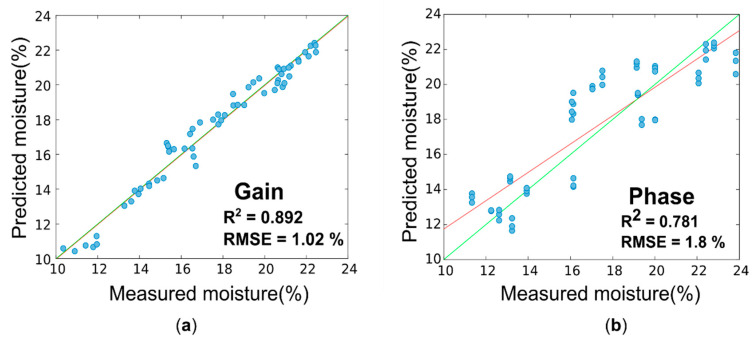
Predicted versus observed values of the moisture content (%) for test set validation of (**a**) gain data and (**b**) phase data. The red line is the line regression for the test samples (so not used for the model creation), whereas the green one is the bisector representing the ideal case. RMSE, root mean square error.

**Figure 12:**
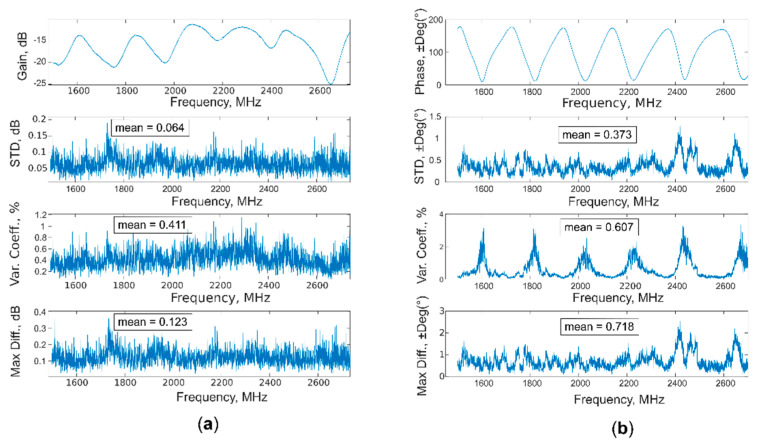
Standard deviation, coefficient of variation, and maximum difference calculated both for (**a**) gain and (**b**) phase, from a total of nine acquisitions on air.

**Figure 13:**
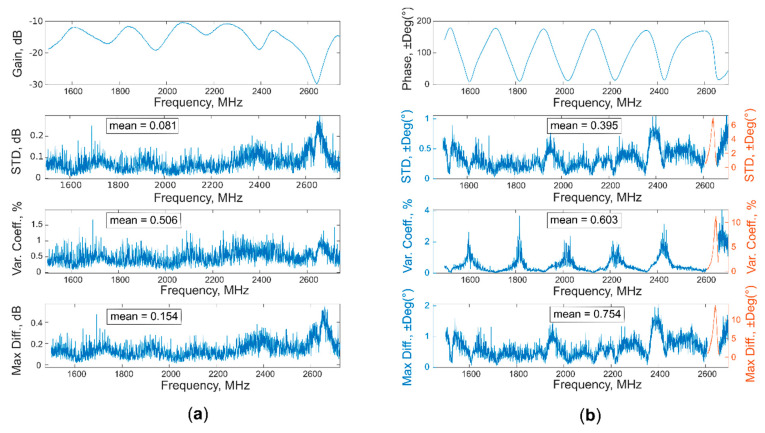
Standard deviation, coefficient of variation, and maximum difference calculated both for (**a**) gain and (**b**) phase, from a total of nine acquisitions on soil.

As possible to observe from Table 2 and Figure 11, the model created with “gain” data is better than the “phase” one, obtaining higher values for R^2^ and RPD, as well as lower values for RMSE, in calibration, cross-validation, and prediction. These results were also supported by the evaluation of accuracy (standard deviation, coefficient of variation, and maximum difference), shown in Figure 12 and Figure 13; the mean values of all three parameters are higher for the phase, both for air and soil acquisitions. Moreover, the value of coefficient of variation presents a peak for every phase region with a high slope, where even a little change in the spectra leads to greater change in the phase values, making the moisture evaluation more unstable.

## 4. Discussion

Comparing the developed system with both traditional and non-invasive alternatives, it is possible to observe several improvements, regarding mainly the spectral acquisition process and the soil moisture prediction ability. With respect to traditional techniques, like TDR and FDR, our system allows a non-invasive measurement, eliminating the instabilities due to the constant contact with the soil and to the presence of air gaps around the probe. These improvements were obtained without diminishing the moisture retrieval ability of the system, tested on a real soil. The obtained RMSEs in calibration (0.7%) and in validation (1.0%) are similar (or even better) to most of the results presented in papers regarding TDR or FDR, such as, for example, the works of [38] (RMSE = 1.26–2.37% in calibration) and [39] (RMSE = 2.0% in validation). Regarding non-invasive systems, the use of an open waveguide leads to a better moisture prediction ability than most of the studies presented in Table 1; the R^2^ and RMSE values are higher than the ones obtained with the fiber spectrometer [16,23] and NIR reflectance sensor [18]. With respect to the papers where the statistical results are better (although never by a large margin), other types of improvements could be found, namely, the use of the PLSR algorithm allows a fast integration of a broad electromagnetic spectrum, with respect to the single frequency used for the linear regression in [19], at the same time being easier to implement than the more complex RFB-ANN analysis used in [21]. Finally, it is also possible to compare these results with the ones reported in a previous work with the same system [4], where an R^2^ of 0.941 and an RMSE of 1.9% were obtained in cross-validation, for similar soil samples characterized by different moisture content. In [4], measurements were done in controlled lab conditions, using homogeneous samples of soil and a constant room temperature, whereas in this work, similar results in terms of prediction were obtained with acquisition conducted on real soil samples. Differences in measurement locations and temperature did not seem to substantially compromise the performance of the technique.

## 5. Conclusions

A proof of concept of contactless open-ended waveguide, designed and developed for soil moisture rapid evaluations, was assessed on a real silt-clay loam soil. “Gain” and “phase” spectra acquired from 1.5 GHz to 2.7 GHz on soil characterized by different moisture contents (from about 9% to 32%) were used to build PLSR models. The best predictive model was obtained starting from “gain” spectra (R^2^ = 0.892, RMSE = 1.0%, RPD = 4.33 for an external test set validation), whereas “phase” spectra did not produce accurate results in terms of moisture content prediction (R^2^ = 0.781, RMSE = 1.8%, RPD = 2.4). As expected, soil temperature can affect both “gain” and “phase” spectral waveforms. However, the multivariate tool appeared to evaluate the variable “moisture content” as the most influential for waveform variance (50.84%, first LV), granting good results in the moisture prediction.

These results are very promising and, in the future, further research could be conducted with the suggested instrumentation and data modelling, trying to refine a more general model based on “gain” data. Furthermore, further developments could be achieved exploring the possibility of predicting moisture at different soil levels, developing a model based on values derived from a combination of “gain” and “phase”, and extending the application of the technique to other kinds of soils and/or chemical soil components, using similar PLSR algorithms, to obtain models for specific soil. Moreover, the performance of the system in different seasons (different temperatures and moisture levels) needs to be investigated and developed, in order to extend the conditions where the acquisition is not distorted. Moreover, the electrical and mechanical design could be improved, decreasing the measuring time, implementing instrumental calibration functions and procedures, and testing the resistance to weather conditions during a prolonged field use.

## Figures and Tables

**Table 1:** Comparison of papers regarding soil moisture evaluation.

Measurement System	Statistical Methods	Laboratory/ Field	R^2^	RMSE (%)	Reference
Fiber spectrometer	PLSR	Field	0.75	2.5	Mouazen et al. (2005)
Fiber spectrometer	PCA	Field	0.65	2.8	An et al. (2011)
NIR reflectance sensor with LED	Not specified	Laboratory	0.64	8.1	Yin et al. (2013)
Bistatic scatterometer	RBF-ANN	Field	----	0.94	Gupta et al. (2016)
Fiber-type VIS/NIR spectrometer	PLSR, LS—SVM, Cubist	Laboratory	0.76 (LS-SVM)	0.45 (LS—SVM)	Morellos et al. (2016)
Open-ended waveguide	PLSR	Laboratory	0.99	0.9	Luciani et al. (2017)
Dipole antenna	K—OPLS	Laboratory	0.97	1.4	Luciani et al. (2018)
NIR detector with laser source	Linear regression	Field	0.89	0.5	Zhou et al. (2019)
Open-ended waveguide	PLSR	Field	0.89	1.0	Present work

PLSR = Partial Least Square Regression, PCA = Principal Component Analysis, RBF-ANN = Radial Basis Function—Artificial Neural Network, LS-SVM = Least Squares Support Vector Machines, K—OPLS = Kernel-based Orthogonal Projections to Latent Structures.

**Table 2:** Partial least squares (PLS) regression models for the prediction of soil moisture content (%) from “gain” and “phase” spectra. RMSE, root mean square error; RPD, residual prediction deviation; LV, latent variable.

Data	LVs	R^2^	RMSE (%)	RPD
Gain—calibration	6	0.958	0.7	
Gain—cross validation	6	0.949	0.7	
Gain—prediction	6	0.892	1.0	4.3
Phase—calibration	5	0.764	1.8	
Phase—cross validation	5	0.741	1.9	
Phase—prediction	5	0.732	1.8	2.4
